# The Path to Driving Aggression and Crash Risk: The Role of Metacognition and Anger Rumination in Anger Expression Among Chinese Drivers

**DOI:** 10.1002/ab.70041

**Published:** 2025-07-02

**Authors:** Chenzhao Zhai, İbrahim Öztürk

**Affiliations:** ^1^ Institute for Transport Studies University of Leeds Leeds UK

**Keywords:** anger rumination, crash risk, driving anger, driving anger expression, metacognition

## Abstract

Driving anger and aggressive anger expression are prevalent in China, leading to road crashes. While potential associations between metacognitive beliefs about worry and control, anger rumination, and anger expression have been reported, limited research focuses on these relationships within the context of driving anger. This study aims to examine the associations between metacognition, anger rumination, driving‐related anger (trait driving anger and aggressive anger expression) and crash risk (traffic penalty points and crash involvement), along with testing the psychometric properties of the Measure for Angry Drivers (MAD) among Chinese drivers. Participants ($M_{age}\;=32.31,\;SD=6.1$) completed the MAD, the short form of the Metacognition Questionnaire (MCQ‐30), the Anger Rumination Scale (ARS), the short version of the Driving Anger Expression Inventory (DAX), and several questions related to their demographic background, traffic violations and crash involvements. A three‐factor structure comprising 23 items of MAD was confirmed (Danger posed by others, Travel delays and Aggression from others), demonstrating good reliability, convergent validity, and criterion validity. Additionally, drivers who were involved in crashes in the past 3 years reported higher total MAD scores. The structural model revealed that trait driving anger influenced anger rumination both directly and indirectly through increased maladaptive metacognitive beliefs. Also, trait driving anger and anger rumination jointly contributed to aggressive anger expression, which in turn significantly predicted crash risk. The current findings demonstrate that the Chinese version of MAD is appropriate for assessing trait driving anger and the necessity of regulating anger rumination and aggressive expressions by modifying maladaptive metacognitive beliefs.

## 1 Introduction

Driving anger has been prevalent in China (Li et al. 2014), and positively associated with driving aggression and road crashes (Zhai and Xi 2023; Zhang et al. 2019). As estimated, dangerous driving and violations (e.g., speeding, illegal overtaking and traffic signal violations) were the main factors of road traffic crashes in the first half of 2020 (The Traffic Management Bureau of the Ministry of Public Security of China 2020).

### 1.1 Driving Anger and Its Measurements

Driving anger has been assessed by using various methodologies. The Driving Anger Scale (DAS) is a self‐report instrument for measuring trait driving anger, a contextual dispositional trait, referring to the propensity to become angry while driving (Deffenbacher et al. 1994). The development of DAS was based on responses from a sample of college students from the United States, resulting in a six factors and 33 items structure (33‐DAS), comprising hostile gestures, illegal driving, police presence, slow driving, discourtesy, and traffic obstructions. Over the past two decades, this scale has been widely used across diverse driving cultures (Deffenbacher et al. 2016). Among these studies, some demographic differences in the DAS have been reported. For instance, drivers with more traffic violations and crashes involvement tend to report higher levels trait driving anger (González‐Iglesias et al. 2012), but the effects of gender was not detected (Deffenbacher et al. 2016). To be noticed, some adaptations have been made to the factorial structure of the DAS when investigating in different driving populations, suggesting the original structure of the 33‐DAS may not be consistent across different cultural contexts.

The factor labeled “progress impedance” (e.g., driving progress being obstructed by other road users) emerged when the original six‐factor structure of the 33‐DAS is failed to be retained (Lajunen et al. 1998; Parker et al. 2002; Sullman 2006). Additionally, although some studies have replicated the six‐factor structure, some factors were found to be highly correlated, e.g., discourtesy and traffic obstructions, r ranging from 0.80 to 0.88 (Li et al. 2014; Sullman et al. 2014). These findings suggest a degree of redundancy and similarity within the factorial structure of the 33‐DAS. Additionally, recent developments in driving may have introduced novel triggers of anger, such as interruptions caused by vehicle technology, which were not accounted for in the 33‐DAS. This also indicates the need to update or extend the scale to assess driving anger.

To answer this need, Stephens et al. (2019) has developed the Measure for Angry Drivers (MAD). The MAD integrates the item pool from diverse sources, such as the DAS and the investigation of social media (Deffenbacher et al. 1994; Stephens et al. 2016), resulting in 23 items with three factors structure, including Danger posed by others, Travel delays, and Aggression from others (r between subscales from 0.61 to 0.68). This scale also shows moderate to strong, and positive relationships with the trait anger scale (Stephens et al. 2019), indicating people who tend to become angry in general circumstances, are also likely to become angry while driving. The factor structure of MAD was recently replicated among Turkish and Australian drivers (Öztürk et al. 2024; Stephens et al. 2024), demonstrating its utility and reliability in assessing trait driving anger among diverse demographic background driving populations in the contemporary driving conditions.

### 1.2 Metacognition, Anger Rumination, and Driving Anger

#### 1.2.1 Understanding Metacognition and Its Role in Psychological Dysregulation

Metacognition is generally defined as “thinking about thinking” (Flavell 1979). It has been suggested that metacognition has at least two functions (Norman et al. 2019), one function is to monitor the current state of whatever cognitive activity we are engaged in (Serra and Metcalfe 2009), and another one is to control our cognition, for example, whether to shifting strategies in the current conditions to efficiently achieve personal goals. Accordingly, metacognition can be understood in two aspects, metacognitive beliefs (also termed metaknowledge) and monitoring of cognition (Schraw et al. 2006; Wells and Cartwright‐Hatton 2004). Metacognitive beliefs are declarative knowledge about cognition, which refers to an epistemological understanding about one's cognition and what factors might influence an individual's performance (Schraw et al. 2006). On the other hand, monitoring of cognition refers to planning of selection and allocation of resources, an awareness of the current state of cognitive experience and task performance, and evaluation of the efficacy of strategies implemented (Flavell 1979; Schraw et al. 2006; Whitebread et al. 2009).

The Self‐Regulatory Executive Function model (S‐REF) was the first to suggest that psychological problems are linked to metacognition (Wells and Matthews 1994). The S‐REF proposes that the emotional disorder/disturbance (e.g., anxiety, depression) is due to the Cognitive Attentional Syndrome (CAS, a style of cognitive‐affective management, such as, perseverative thinking (e.g., rumination), thoughts suppression, avoidance, and threat monitoring) (Wells and Matthews 1994), which is activated and maintained by specific metacognitive beliefs (Wells and Matthews 1996). The Metacognition Questionnaire (MCQ‐30) was developed to assess different types of metacognitive beliefs (Wells and Cartwright‐Hatton 2004), including (1) positive beliefs about worry, stressing on the value of using worry to solve issues, for example, “I need to worry to work well.” (2) negative beliefs about uncontrollability and danger, emphasizing on the uncontrollability and danger of thoughts, for example, “I could make myself sick with worrying.” (3) cognitive confidence, referring to the efficacy of one's attention and memory, for example, “I have little confidence in my memory for actions.” (4) cognitive self‐consciousness, reflecting the extent to which an individual focuses on their thinking process, for example, “I am constantly aware of my thinking.” and (5) need to control thoughts, pertaining to beliefs about the necessity for controlling thoughts, for example, “If I did not control a worrying thought, and then it happened, it would be my fault.” (Kuhn and Dean, Jr. 2004; Wells and Cartwright‐Hatton 2004).

Some research revealed that individuals retaining more maladaptive metacognitive beliefs (e.g., positive beliefs about worry, negative beliefs about uncontrollability and danger, cognitive confidence, and need to control thoughts) reported higher levels of negative emotions, such as anxiety and anger (Caselli et al. 2017; Cook et al. 2014; Kara et al. 2023). In contrast, the cognitive self‐consciousness shows no association with anxiety and depression (Cook et al. 2014; Tajrishi et al. 2011), but it impairs the capacity of emotional regulation (Mansueto et al. 2022).

Anger rumination can be viewed as a type of a cognitive dysregulation associated with emotional experience (Gratz and Roemer 2004; Mansueto et al. 2024), which is a repetitive and dysfunctional cognition of anger‐related experiences, e.g., repetitively recalling angry feelings, angry thoughts, and angry reflections (Anestis et al. 2009). Previous studies indicated that higher levels of anger rumination after the provocation could lead to a higher intensity of anger and aggression (Anestis et al. 2009; Salguero et al. 2020). As discussed above, metacognitive beliefs have been considered be important antecedents of the CAS. Rumination as a typical component of the CAS, have been found to be significantly and positively predicted by metacognitive beliefs (Salguero et al. 2020), suggesting these beliefs contribute to the persistence and intensification of anger rumination (Caselli et al. 2017; Krans et al. 2014).

#### 1.2.2 The Potential Role of Metacognition in Aggressive Anger Expression While Driving

Previous studies have demonstrated that drivers with higher trait driving anger exhibit maladaptive cognitive patterns, as characterized by reduced anger‐control thoughts (Nesbit and Conger 2011) and a greater tendency to ruminate anger‐provoking experience (Suhr 2016). These cognitive patterns are further associated with increased aggressive anger expression while driving (Deffenbacher et al. 2004). Such findings also suggest that individuals with higher anger propensities may experience difficulties in managing their cognitive processes (i.e., metacognition), which in turn impairs their capacity to regulate anger‐related behavioral responses on the road. Specifically, these regulatory difficulties may stem from preservative thinking (e.g., worry and rumination), heightened self‐focused (e.g., continually threat monitoring), and maladaptive coping strategies (e.g., suppression of thoughts). These factors collectively contribute to enhanced negative thoughts and impaired cognitive flexibility (Wells 2008; Wells and Matthews 1996), making it more challenging to disengage from anger‐related experiences and adopt adaptive emotional regulation strategies.

Despite growing interest in the cognitive mechanisms underlying driving anger, the role of metacognition remains underexplored. To date, to the best of our knowledge, only one study has explored the role of metacognition in the field of driving anger (Love et al. 2022). They found that drivers with more negative metacognitive beliefs about rumination (e.g., “Ruminating about my problems is uncontrollable.”) could lead to frequent engagement in anger rumination which results in aggressive anger expression while driving. Although this study provides important insights, the impacts of metacognitive beliefs on anger rumination might not be fully captured. More recent evidence shows that other metacognitive beliefs such as beliefs about need to control, as well as uncontrollability and danger, are also significantly associated with rumination tendencies (Mansueto et al. 2022). It is possible that individuals with higher trait driving anger retained more thoughts of control, also frequently engage in excessive monitoring and repetitive thinking, which in turn impedes in using adaptive emotion regulation strategies (Spada et al. 2008). Moreover, it has been proposed that one's reactions to their own thoughts might determine the extent to which these thoughts influence behaviors (Hamonniere and Varescon 2018; Petty et al. 2007). This indicates that such metacognitive beliefs may not only sustain anger rumination but could also play a direct role in influencing aggressive anger expression. However, the extent to which metacognitive beliefs related to aggressive anger expression in the driving anger context remains unclear, as Love et al. (2022) only provided correlational findings without testing directional path relationships between these constructs. If a significant relationship between metacognitive beliefs, anger rumination, and aggressive anger expression could be established, this would have important implications for developing intervention strategies targeting metacognitive processes to manage anger expression in driving contexts.

### 1.3 The Present Study: Study Rationale, the Proposed Theoretical Model, and Study Aims

As discussed in Section 1.1, the MAD represents a more contemporary instrument as compared to the DAS for assessing trait driving anger. However, its psychometric properties and potential differences across demographic groups have not been evaluated in the Chinese context. Therefore, there is an essential need to assess the factorial structure, reliability and validity of MAD, along with an examination of potential demographic differences in MAD, which might contribute to a broader international perspective on its applicability.

**Figure 1:**
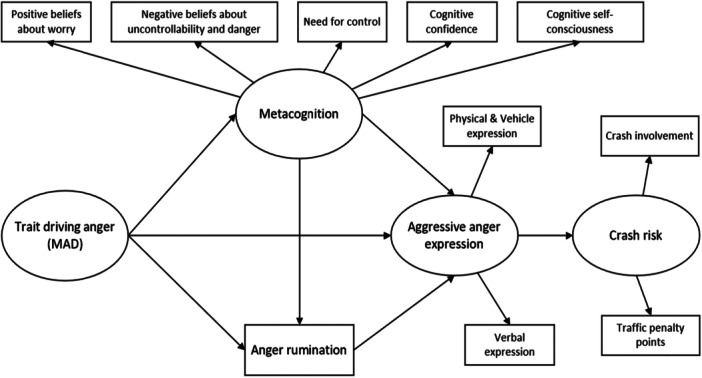
The proposed theoretical framework in the present study.

Moreover, existing literature indicates that Chinese drivers not only exhibit aggressive behaviors in response to anger‐provoking situations (Li et al. 2014; Zhai et al. 2023), but they also demonstrate negative cognitive patterns such as an increased sense of control when encountering such situations (Zhai et al. 2024). Also, some studies have found that Chinese people tend to ruminate about anger experiences more frequently than individuals in Great Britain (Maxwell et al. 2005). These findings may suggest an essential need regarding focusing cognitive regulation in reducing aggressive anger expression while driving among Chinese drivers. However, to the best of our knowledge, limited understanding has been provided regarding how to regulate dysfunctional cognitive responses (e.g., anger rumination) associated with driving anger. To address this gap, the theoretical model is proposed as shown in Figure 1.

Herein, metacognition is regarded as a latent construct which reflected by multifaced of metacognitive beliefs as measured by MCQ‐30 (Wells and Cartwright‐Hatton 2004). It should be noted that only aggressive forms of anger expression while driving were included in the theoretical model, not only due to their potential positive associations with metacognition as reported by literature, but it also poses a significant threat to road safety (Deffenbacher et al. 2002). Considering the direct link between trait driving anger and aggressive anger expression (Deffenbacher et al. 2003), as well as the potential associations between metacognitive beliefs and aggressive anger expression (Demir et al. 2016; Love et al. 2022), the construct of metacognition is considered as a mediator between trait driving anger and aggressive anger expression. Additionally, prior research has demonstrated that anger rumination mediates the relationship between anger propensity and aggressive driving (Suhr 2016), it is considered as a mediator in the present study. Importantly, in alignment with the proposition of S‐REF model, metacognition is an important antecedent of maladaptive cognitive patterns (e.g., rumination), the path between them is indicated within the proposed model as well.

Lastly, road collisions pose a threat to public safety and national development (World Health Organization 2024), the present study considers crash risk as a critical outcome variable of aggressive anger expression (Demir et al. 2016), which may concreate the impacts of the present study in both theoretical and practical implications. The crash risk was defined based on Simer et al. (2005); Zhang et al. (2019), because the reflection of different crash‐related conditions for comprehensively measuring crash risk is necessary (Dahlen et al. 2012).

In specific, the study aims of the present study are listed as follows:

1. To assess the psychometric properties of the MAD and examine demographic differences in MAD among Chinese drivers.
2. To explore the associations among metacognition, anger rumination, driving‐related anger (trait driving anger and aggressive anger expression), and crash risk among Chinese drivers.

## 2 Methodology

### 2.1 Data Collection and Participants

Participants were recruited with the assistance of a Chinese survey company: Wen Juan Xing (www.wjx.cn), which operates the largest online survey platform in China, and other Chinese researchers also used this platform, such as Zhang et al. (2019). The criterion for participants' recruitment was having a valid Chinese driving license. Participants were informed that their participation was voluntary and that their responses would be kept anonymous. All participants were compensated with 15 RMB (approximately 2.2$) for their time. This study was approved by the ethical committee of the University of Leeds (BESS + FREC 2023‐0692‐869).

Previous studies, as a rule of thumb, suggested the sample size for Confirmatory Factor Analysis (CFA) should be at least 10 times the total number of measured indicators of the scale (Nunnally and Bernstein 1967; Wang and Wang 2019), which implies a minimum requirement of 230 participants. In addition, we have also estimated the required sample size for the proposed structural model based on the Root Mean Squares Error of Approximation (RMSEA) of 0.07, α (Error Type I)=0.05,β (Error Type II)=0.20,Degress of Feedom (DF)=59 (Hair et al. 2010; Kim 2005), suggesting that a sample size of at least 113 participants is sufficient without the consideration of dropout rate. However, in consideration of invalid responses given that previous experience with this online survey platform, there is an approximately 35% rate of invalid responses (e.g., failed to respond to attentional check), we aimed for a higher sample size (minimum 350).

In total, 1035 participants were recruited, but 421 individuals' responses were regarded as valid after the data filtration (i.e., attentional questions check and too short response time [*n* = 611], outlier age and driving experience [Z score > 3.5, *n* = 2], invariability of the answer [*n* = 1]). Of the final sample, 56.5% were males, the mean age was 32.31 (range from 20 to 55, SD = 6.1) and the mean of annual mileages (km) was 7679.18 (range from 2 to 30,000, SD = 6549.45). Despite the attrition, the retained sample remained broadly representative of the general Chinese driving population in terms of age and gender distribution (The Traffic Management Bureau of the Ministry of Public Security of China 2021), and geographic coverage, with participants recruited from 30 provinces (accounting for 88% of all provincial‐level regions in China). More detailed demographic information on participants can be found in Table 1.

**Table 1:** Participants' demographic information (*N* = 421).

Variables	*N*	Proportion
Gender		
Male	183	43.5%
Female	238	56.5%
Length of the driving license		
1–3 years	91	21.6%
4–6 years	131	31.1%
7–10 years	139	33.0%
＞ 10 years	60	14.3%
Annual mileage		
< 2000 km	123	29.2%
2001–5000 km	58	13.8%
5001–10000 km	129	30.6%
10001–20000 km	101	24.0%
> 20001 km	10	2.4%
Traffic penalty points received in the last year		
0	231	54.9%
1–2	9	2.1%
3–6	41	9.7%
7–12	140	33.3%
Crashes involved in the past 3 years		
0	315	74.8%
1	78	18.5%
2	22	5.2%
≥ 3	6	1.4%

### 2.2 Measurements

**Demographic information:** Participants were asked to report their age, gender, annual driving experience (in kilometers), traffic penalty points received in the last year and crashes involved in the last 3 years.

**Measure for Angry Drivers:** The MAD is a self‐reported tool for assessing people's tendency to become angry while driving (Stephens et al. 2019). In its original version, MAD has 23 items and three broad anger‐provoking categories: Danger posed by others (12 items), Travel delays (7 items) and Aggression from others (4 items). Participants were required to report the extent of anger if they encounter a specific situation (e.g., “Someone beeps at you without reason.”) on a 5‐point scale from (1 = “Not angry at all” to 5 = “Extremely angry”). As the MAD has not been formally assessed in China, the measure is adapted to Chinese in this study. The translation and back‐translation procedure were conducted based on three independent psychologists who are proficient in both Chinese and English. Some items were slightly changed to align with the Chinese driving environment, for example, item 7 was revised from “A jaywalking pedestrian crosses in front of you forcing you to brake.” to “Jaywalking pedestrians and cyclists cross in front of you forcing you to brake.” Higher scores indicate a higher level of trait driving anger. The overall Cronbach's alpha of the MAD was 0.93 in the present study.

**The short form of the Metacognition Questionnaire:** To assess drivers' metacognitive beliefs, the Chinese version of MCQ‐30 was used in the present study (Zhang et al. 2020). This instrument has 30 items and assesses metacognitive beliefs in five aspects such as positive beliefs about worry [POS] (e.g., “I need to worry, to work well.”); negative beliefs about uncontrollability and danger [NEG] (e.g., “My worry could make me to mad.”); cognitive confidence [CC] (e.g., “I have little confidence in my memories and actions.”); need for control [NC] (e.g., “I should be in control of my thoughts all of the time.”); and cognitive self‐consciousness [CSC] (e.g., “I pay close attention to the way my mind works.”). Participants needed to rate each item on a 4‐point scale (1 = “Do not agree” to 4 = “Agree too much”). The Chinese MCQ‐30 shows acceptable internal consistency in the present study (Cronbach's alpha = 0.66–0.83). Higher scores of these dimensions reflect more maladaptive metacognitive beliefs (Chen et al. 2021).

**The Anger Rumination Scale (ARS):** The ARS is devised to measure an individual's tendency to focus attention on anger experience and the cause and consequence of the anger episode, for example, “I ruminate about my past anger experiences.” (Sukhodolsky et al. 2001). The Chinese version of ARS was employed in this study, consisting of 19 items (Wang et al. 2018), showing excellent reliability (overall Cronbach's alpha = 0.95). Items were rated on a 4‐point scale (1 = “Never” to 4 = “Always”). Higher scores suggest a greater degree of ruminating anger‐related experience. The Cronbach's alpha was 0.93 in the present sample.

**The Driving Anger Expression Inventory (DAX):** Sullman and Stephens (2014) developed the short form of the DAX, increasing its conjunct‐ability with other scales when investigating forms of drivers' anger expressions. Recently, Zhai et al. (2023) assessed the short version of DAX among the Chinese sample, revealing 11 items and three‐factor solutions (Adaptive Expression [AE], Verbal Expression [VE], and Physical and Vehicle Expression [PVE]). Participants rated each item on a 4‐point scale (1 = “Almost never” to 4 = “Almost always”). This instrument shows acceptable reliability among the present sample (Cronbach's alpha = 0.71–0.81). Higher scores indicate the propensity to express anger in either adaptive or aggressive ways while driving.

### 2.3 Data Analysis

Data analyzes were conducted in SPSS 27.0 and Mplus 7.0. Before the main analyzes, the univariate normality of continuous variables was assessed through skewness and kurtosis statistics. All variables showed acceptable levels (|skewness| < 3, |kurtosis| < 7), indicating approximate normality (Byrne 2010). To explore differences in MAD scores across demographic groups, a series of Analysis of Covariance (ANCOVA) tests were conducted, controlling for relevant covariates (traffic penalty points and crash involvement).

CFA with Mean‐adjusted Maximum‐Likelihood approach was directly performed on MAD, to examine whether the original structure of MAD was applicable in the Chinese context, implying the expectation that the structure of the scale is appropriate among research populations (Flora and Flake 2017). Internal consistency of the MAD was assessed using Cronbach's alpha, and convergent validity was evaluated through Average Variance Extracted (AVE), with a threshold of 0.40 considered acceptable (Fornell and Larcker 1981).

Furthermore, Structural Equation Modeling (SEM) was employed to test the relationships among metacognitive beliefs, anger rumination, driving‐related anger, and crash risk. The SEM was estimated using Maximum Likelihood with bootstrapping (2000 samples) to account for the potential effects of multivariate non‐normality (Hair et al. 2019). The model fit for both CFA and SEM was assessed using multiple indices. Specifically, Comparative Fit Index (CFI) of 0.90 or higher and a RMSEA of 0.06 or lower were considered indicators of excellent fit, while an RMSEA upper bound of the 90% Confidence Interval (CI) not exceeding 0.08 was deemed acceptable (Hu and Bentler 1999).

## 3 Results

### 3.1 The Factorial Structure of the MAD and Its Psychometric Properties

The factorial structure of the Chinese MAD is shown in Table 2. The initial CFA model, without any correlated errors, indicating an acceptable fit (CMIN/DF = 2.62, CFI = 0.89, RMSEA = 0.064). However, it was suggested that five error pairs were allowed to covary, because all modification indices (MIs) were over 15 (Ge et al. 2015). Specifically, these included e1–e2 [MIs = 32.441], e2–e5 [MIs = 31.411], e4–e5 [MIs = 16.419], e13–e14 [MIs = 25.176], e20–e21 [MIs = 23.369]. This might be due to similar wording and response style and potentially share an unobserved and exogenous common factor, which has not been captured by the current model (Brown 2015). Following these modifications, the revised model demonstrated an improved fit, confirming that the 23 item and three factors solution MAD is suitable for Chinese drivers (CMIN/DF = 2.17, CFI = 0.93, RMSEA = 0.053 with 90% CI [0.046–0.059]). These factors were respectively labeled as “Danger posed by others” (referring to situations where anger is caused by other road users posing threats), “Travel delays” (referring to situations where anger is provoked by travel impediments) and “Aggression from others” (referring to situations in which anger is evoked in response to aggressive actions from other road users). In addition, the Corrected Item to Total Correlations (CITC) ranged from 0.49 to 0.66, indicating the relevance and importance of items retained in the Chinese version of the MAD. Moreover, the convergent validity of three subscales was demonstrated by the AVE, in which “Travel delays” and “Aggression from others” exceeded the threshold, but the AVE of “Danger posed by others” was 0.39, approximate to the cutoff value of 0.40.

**Table 2:** Factorial structure of the Chinese version of the MAD.

Items		Danger posed by others (*M* = 3.49, SD = 0.69, α = 0.89, AVE = 0.39)	Travel delays (*M* = 2.42, SD = 0.79, α = 0.85, AVE = 0.45)	Aggression from others (*M* = 3.61, SD = 0.90, α = 0.83, AVE = 0.53)	MAD Total (*M* = 3.19, SD = 0.65, α = 0.93)	Skewness	Kurtosis
1	Someone pulls out right in front of you without looking.	0.52			3.63 (0.96)	−0.44	−0.13
2	Someone moves in front of you suddenly and without leaving enough room, forcing you to brake hard.	0.56			4.16 (0.98)	−1.23	1.11
3	Another driver causes a near miss with your vehicle.	0.53			3.90 (0.99)	−0.66	0.01
4	Someone cuts in right in front of you forcing you to brake.	0.60			3.77 (0.98)	−0.53	−0.31
5	Someone does an illegal U turn in front of you, forcing you to brake hard.	0.58			4.01 (0.99)	−0.95	0.49
6	A driver fails to indicate at an intersection, roundabout, or when making a turn in front of you.	0.62			3.23 (0.97)	−0.25	−0.42
7	Jaywalking pedestrians and cyclists cross in front of you forcing you to brake.	0.54			3.69 (1.10)	−0.52	−0.50
8	A driver fails to give way to you when supposed to e.g., at an intersection, parking or give way sign.	0.68			3.06 (0.97)	−0.22	−0.34
9	When you are trying to overtake another driver he/she speeds up.	0.70			2.65 (1.12)	0.22	−0.69
10	A driver ahead of you is straddling two lanes.	0.69			3.43 (1.09)	−0.26	−0.70
11	Someone pulls out right in front of you when there is no‐one behind you.	0.70			3.16 (1.11)	−0.18	−0.68
12	When you are trying to merge, other drivers do not give way (preventing you merging).	0.66			3.19 (1.11)	−0.18	−0.66
13	You encounter road works and detours.		0.67		1.76 (0.95)	1.35	1.45
14	You see a flash and are unsure whether you have been photographed by a hidden speed camera.		0.68		2.14 (1.01)	0.64	−0.11
15	You have difficulty getting something you are using to help you drive to work properly or the way you want it to (e.g. vehicle/phone map navigations).		0.65		2.86 (1.03)	−0.05	−0.63
16	You have a green left turn arrow; however, a driver ahead is traveling straight and blocking the turn.		0.67		2.85 (1.14)	0.10	−0.82
17	You are driving behind a large vehicle and you cannot see around it.		0.70		2.41 (1.14)	0.46	−0.63
18	Someone in front of you does not move off straight away when the light turns to green.		0.72		2.46 (1.15)	0.43	−0.63
19	You are stuck in peak hour traffic.		0.61		2.51 (1.15)	0.30	−0.76
20	Someone shouts at you about your driving.			0.72	3.68 (1.08)	−0.66	−0.14
21	Someone makes a rude gesture towards you about your driving.			0.70	3.82 (1.08)	−0.78	−0.13
22	Another driver indicates anger/hostility when you do a perfectly legal maneuver.			0.73	3.56 (1.13)	−0.66	−0.24
23	Someone beeps at you without reason.			0.76	3.39 (1.11)	−0.26	−0.70

### 3.2 Intercorrelation Among Variables

Pearson correlations (see Table 3) were performed to probe if there were any associations among driving‐related anger, metacognitive beliefs, anger rumination and crash risk.

**Table 3:** Pearson intercorrelation among variables.

Variables	1	2	3	4	5	6	7	8	9	10	11	12	13	14	15	16	17	18
1 Age																		
2 Gender (0=Males)	**−0.129** *																	
3 Years of driving	**0.668** **	**−0.235** **																
4 Annual (km)	**0.181** **	**−0.341** **	**0.206** **															
5 Points	**0.097** *	**−0.163** **	0.078	0.092														
6 Crashes	0.053	−0.083	0.052	0.033	**0.296** **													
7 POS	0.005	−0.016	−0.004	0.008	0.025	**0.106** *												
8 NEG	**−0.102** *	**0.122** *	**−0.105** *	−0.072	0.029	0.042	**0.217** **											
9 CC	−0.020	**0.135** **	−0.046	**−0.134** **	−0.028	0.024	**0.139** **	**0.531** **										
10 NC	−0.019	−0.061	−0.049	0.009	0.075	0.054	**0.351** **	**0.454** **	**0.332** **									
11 CSC	**−0.118** *	−0.056	−0.071	**0.099** *	0.093	0.055	**0.335** **	0.040	**−0.114** *	**0.174** **								
12 AE	0.056	0.029	0.001	**0.121** *	0.038	**−0.127** **	−0.026	**−0.117** *	**−0.131** **	−0.047	**0.160** **							
13 VE	0.044	**−0.106** *	0.014	−0.024	**0.131** **	**0.203** **	**0.193** **	**0.178** **	0.071	**0.154** **	0.087	**−0.272** **						
14 PVE	−0.067	**−0.113** *	0.014	0.068	0.065	**0.105** *	**0.192** **	**0.240** **	**0.157** **	**0.246** **	0.012	**−0.188** **	**0.428** **					
15 MAD‐Danger	0.053	0.001	0.043	0.037	0.073	**0.096** *	**0.158** **	**0.168** **	0.095	**0.160** **	0.065	**−0.220** **	**0.390** **	**0.242** **				
16 MAD‐Travel	−0.015	0.005	0.010	−0.007	**0.107** *	0.071	**0.224** **	**0.244** **	**0.167** **	**0.285** **	0.028	**−0.253** **	**0.362** **	**0.421** **	**0.646** **			
17 MAD‐Aggression	−0.068	0.031	−0.025	0.059	0.056	**0.132** **	0.095	**0.173** **	**0.173** **	**0.132** **	0.042	**−0.207** **	**0.370** **	**0.232** **	**0.599** **	**0.489** **		
18 Total MAD	−0.009	0.010	0.022	0.033	0.093	**0.110** *	**0.192** **	**0.224** **	**0.155** **	**0.225** **	0.056	**−0.264** **	**0.436** **	**0.343** **	**0.931** **	**0.841** **	**0.748** **	
19 Anger rumination	−0.089	0.055	−0.076	−0.024	0.085	**0.128** **	**0.224** **	**0.566** **	**0.400** **	**0.362** **	**0.111** *	**−0.166** **	**0.371** **	**0.317** **	**0.317** **	**0.325** **	**0.314** **	**0.369** **

Abbreviations*:* AE, adaptive expression; CC, confidence consciousness; Crashes, crashes involvement during the past three years; CSC, cognitive self‐consciousness; NC, need for control; NEG, negative beliefs about uncontrollability and danger; POS, positive beliefs about worry; Points, traffic penalty points received in the last year; PVE, physical and vehicle expression; VE, verbal expression.

* *p*< 0.05

** *p*< 0.01.

It was observed that males tend to be deducted traffic penalty points in the last year. Male drivers also showed more maladaptive beliefs in negative beliefs about uncontrollability and danger and cognitive confidence. Notably, some maladaptive metacognitive beliefs were significantly related to anger rumination, such as negative beliefs about uncontrollability and danger and need for control. These maladaptive beliefs were also positively related to aggressive anger expression, such as negative beliefs about uncontrollability and danger and VE; need for control and PVE, implying maladaptive thoughts could lead to driving aggression.

Moreover, three dimensions of MAD were positively correlated with VE and PVE, but negatively associated with the AE, indicating the criterion validity of the Chinese version MAD. Besides, it was observed that the subscales “Danger posed by others” and “Aggression from others” were positively related to crash involvement in the past 3 years. Notably, the MAD was also positively associated with anger rumination, indicating that drivers with higher levels of trait driving anger may be more prone to engaging in anger rumination.

### 3.3 Demographic Differences in MAD

To examine demographic differences in the Chinese version of MAD, several ANCOVAs were performed with the statistical effects of demographics background being controlled for (see Table 4). The assumption of homoscedasticity between covariates and independent variables was found to be satisfied (p>0.05).

**Table 4:** MAD difference across the demographic background.

Variables	Gender		*Significance*	Traffic violation (Penalty points)		*Significance*	Crash involvement		*Significance*
Category	Males	Females	*p*	Deducted	Non‐deducted	*p*	Involved	Non‐involved	*p*
MAD‐Danger	3.49 (0.74)	3.49 (0.65)	*0.749*	3.45 (0.68)	3.54 (0.69)	*0.195*	**3.44 (0.73)**	**3.63 (0.52)**	***0.012*** [Link], *
MAD‐Travel	2.42 (0.86)	2.43 (0.74)	*0.621*	2.36 (0.79)	2.50 (0.79)	*0.077*	2.38 (0.81)	2.54 (0.71)	*0.080*
MAD‐Aggression	3.58 (0.95)	3.64 (0.85)	*0.385*	3.57 (0.93)	3.67 (0.85)	*0.588*	**3.53 (0.94)**	**3.85 (0.71)**	***0.001*** ***
MAD total	3.18 (0.71)	3.19 (0.60)	*0.561*	3.14 (0.64)	3.24 (0.66)	*0.254*	**3.14 (0.68)**	**3.34 (0.53)**	***0.018*** [Link], *

*Notes:* Covariates: Traffic penalty points and crash involvement for gender, crash involvement for traffic penalty points, traffic penalty points for crash involvement.

* *p* < 0.05

** *p* < 0.01

*** *p* < 0.001.

As expected, no gender differences were found across all scales of MAD. Similarly, there were no significant differences between traffic rule violators and non‐traffic rule violators in terms of their scores rated on MAD. Notably, drivers involved in crashes reported higher total scores of MAD, especially in subscales “Danger posed by others” and “Aggression from others.”

### 3.4 The Associations Among Trait Driving Anger, Metacognition, Anger Rumination, Aggressive Anger Expression and Crash Risk

**Figure 2:**
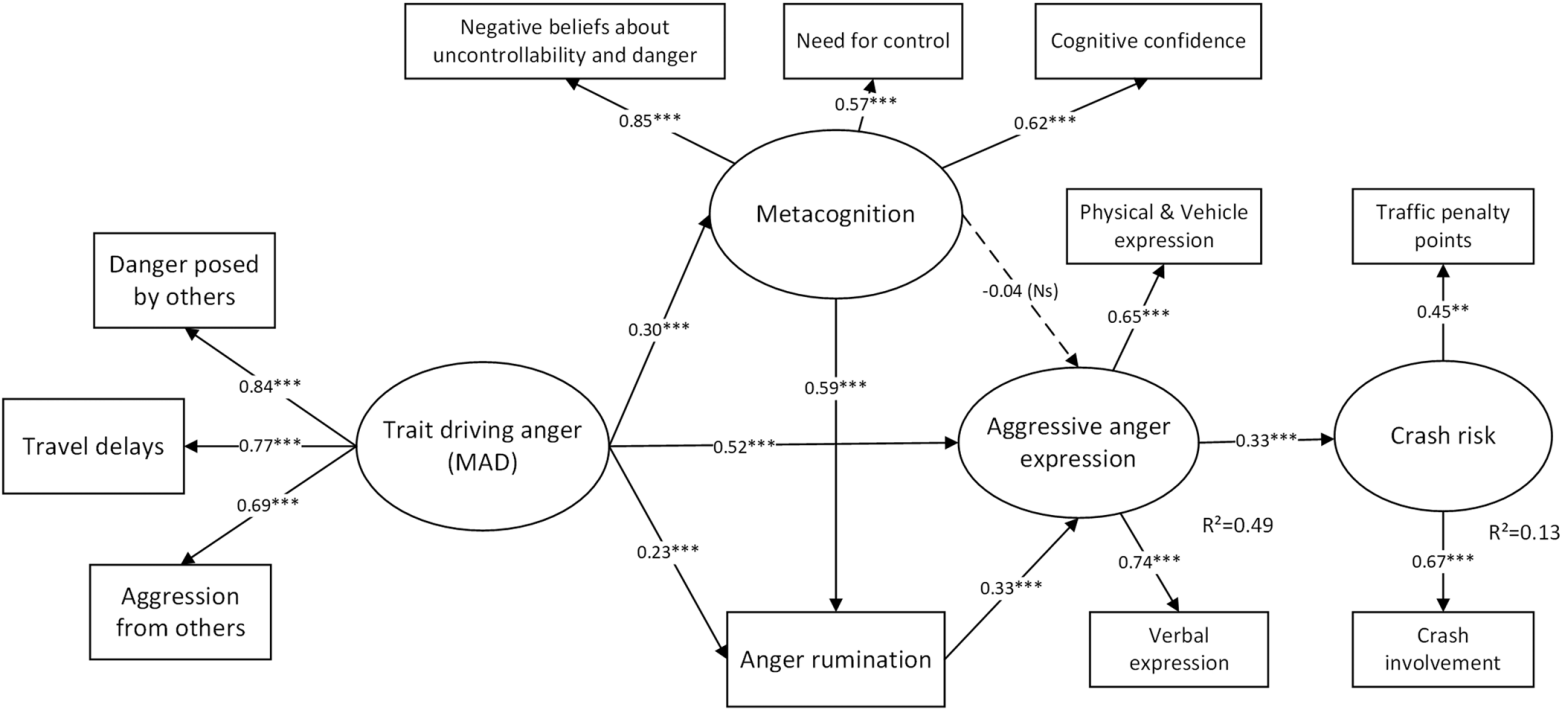
Results of SEM among metacognition, anger rumination, driving‐related anger, and crash risk. CMIN/DF = 2.27, CFI = 0.96, RMSEA = 0.055, 90%CI [0.040–0.069], ** p < 0.01, *** p < *0.001, Ns= Not significant*.

To explore the relationships between maladaptive metacognitive beliefs and anger rumination, driving‐related anger and crash risk, a SEM approach was employed based on the proposed model (Figure 1). Regarding the metacognition construct, two dimensions of the metacognitive beliefs were excluded (CSC and POS), because of the lower factor loading (less than 0.50), suggesting they were not well‐explained by the latent factor of metacognition. Results showed that the model fit was acceptable, illustrated in Figure 2 (standardized coefficients).

As expected, trait driving anger (measured by the MAD) was significantly and positively associated with aggressive anger expression, with a direct effect accounts for 78.8% of the total effect (β=0.52, 95%CI[0.40,0.65]). In addition to this direct pathway, angry disposition (trait driving anger) influenced aggressive anger expression through two indirect pathways. The first indirect pathway through the anger rumination was significant (β=0.08, 95%CI[0.03,0.11]), accounting for 12.1% of the total effect of trait driving anger on aggressive anger expression. This suggests that individuals with high trait driving anger are more likely to ruminate about anger‐provoking events, which in turn enhances aggressive anger expression. Moreover, another indirect pathway via both metacognition and anger rumination, was also significant (β=0.06, 95%CI[0.05,0.06]), contributing 9.1% of the total effect. This path relationship indicates that drivers who are prone to experience anger, tend to retain maladaptive metacognitive beliefs, which promote anger rumination, and ultimately lead to increased driving aggression.

However, the direct relationship between metacognition and aggressive anger expression was not significant, suggesting these metacognitive beliefs did not directly determine aggressive behaviors. Furthermore, a significant, positive, and moderate association was observed between aggressive anger expression and crash risk, suggesting that drivers who frequently express anger aggressively are at a higher risk in traffic penalties and crash involvement. Overall, the proposed model shows considerable explanatory power in aggressive anger expression and crash risk.

## 4 Discussion

The present study assessed and validated the psychometric properties of the MAD among Chinese drivers and examined the associations between metacognitive beliefs, anger rumination, driving‐related anger, and crash risk in the context of driving anger in China.

### 4.1 Psychometric Properties of the Chinese MAD

The Chinese version of MAD retained the full items and factorial structure as the original version of MAD (Stephens et al. 2019). Three factors accordingly assessed three common anger‐provoking sources: “Danger posed by others,” “Travel delays,” and “Aggression from others.” The factorial structure of the Chinese MAD shows acceptable reliability and validity. Compared to the previous findings by Zhang et al. (2018), it appears that drivers in China are increasingly prone to be angry in the contemporary driving environment, which might be due to the growing number of vehicles and drivers on the road^1^, intensified competitive driving (Li et al. 2016), and traffic conflicts between road users, e.g., the right of the way (Huo et al. 2023). Some demographic differences in MAD were also detected in the present study. For instance, drivers involved in crashes reported higher levels of trait driving anger, particularly in the “Danger posed by others” and “Aggression from others” sub‐scales. This suggests that anger triggered by perceived external threats or hostile behaviors may be more closely associated with crash occurrence. Consistent with the previous studies (Li et al. 2014; Zhang et al. 2018), no gender difference was found in trait driving anger as measured by the MAD. Interestingly, the present study found that drivers with and without traffic violations exhibited similar levels of trait driving anger, as measured by the MAD. This suggests that the association between traffic penalty points and trait driving anger may be more complex than what was observed in the present study. There has been shown that driving behaviors are an important mediator between anger propensity and traffic violations (Zhang et al. 2019). It is also possible that individuals prone to experience anger may not necessarily engage in behaviors that result in formal penalties, because of the effects of social norm (Fruhen and Flin 2015), e.g., negative attitudes toward risky driving. Overall, the present study provides internationally empirical evidence to support that the MAD is a reliable tool for assessing trait driving anger within contemporary driving contexts (Stephens et al. 2024).

### 4.2 The Associations Between Metacognition, Anger Rumination, Driving‐Related Anger and Crash Risk

Another important aim of the present study is to extend the understanding of the domain of driving anger, based on perspectives of metacognitive beliefs about worry and control. According to the current results, the proposed model and the amount of variance explained demonstrated that it fits the data well among Chinese drivers, indicating the proposed model might be useful for capturing underlying mechanisms of metacognition, anger rumination, driving‐related anger, and crash risk.

As expected, a significant and direct path was found between trait driving anger and aggressive anger expression, demonstrating the predominant role of anger tendencies in raged actions (Deffenbacher et al. 2003). In addition, the mediating role of anger rumination in trait driving anger and anger rumination was found among Chinese drivers, coinciding with other studies (Li and Xia 2020; Suhr 2016). However, some metacognitive beliefs (i.e., NEG, NC, and CC) were not found to be directly related to aggressive anger expression. Instead, their impacts were mediated through anger rumination, aligning with the findings from recent research (Salguero et al. 2020). This possibly suggests that, in the context of driving anger, individuals with high trait anger tend to engage in rumination after encountering anger‐inducing stimuli, and that biased and maladaptive metacognitive processes may further exacerbate the extent of such rumination.

Importantly, Chinese drivers with the higher trait driving anger exhibited more maladaptive metacognitive beliefs, including a sense of control (e.g., “I should be in control of my thoughts all of the time.”), poor confidence in their memory (e.g., “I have little confidence in my memory for actions.”), and negative thoughts about uncontrollability and danger (e.g., “I cannot ignore my worrying thoughts.”). These three aspects of metacognitive beliefs were significantly and positively correlated with a moderate to strong magnitude. It is possible that a high need for control thoughts combined with low confidence in memory may promote repetitive thinking about the initial anger‐triggering event. However, without targeted interventions, these maladaptive metacognitive patterns may reinforce each other, creating a vicious cycle that perpetuates anger rumination (Evli and Şimşek 2021). Also, these findings highlight the importance of enhancing self‐regulation when dealing with maladaptive and angry thoughts, because there has been demonstrated that when facing anger‐induced situations, the accessibility and maintenance of negative information (e.g., negative appraisal and anger rumination) might be determinative for promoting aggression (Denson 2013).

Notably, some metacognitive beliefs were excluded in the structural model (POS and CSC), suggesting they may be less relevant in the context of driving anger among Chinese drivers. In contrast to NEG, the POS refers to metacognitive optimism (e.g., “Worrying helps me to cope.”), the belief that worry can be beneficial for managing future threats. However, in stressful driving situations, anger‐provoking situations in particular, such beliefs may have limited impacts. It has been well‐acknowledged that anger is always elicited by a perceived conflict of personal goals (Smith and Lazarus 1993). When drivers are exposed to immediate threats, delays, or discourteous behaviors, the POS may not significantly lead to emotional responses and behavioral changes (Penney et al. 2013).

Similarly, CSC refers to the extent to which individuals focus attention on their own thoughts and cognitive processes, but it was neither significantly correlated with trait driving anger nor with aggressive anger expression. This suggests that merely being aware of one's cognitive activity does not inherently shape how one experiences or expresses anger in the context of driving anger. Probably, CSC may act as an antecedent of appraisal which might be more predictive to behavioral tendencies, e.g., driving anger expression (Gilbert et al. 2013).

The present findings demonstrate that metacognitive beliefs about worry and control contribute to anger rumination in the context of driving anger, in addition to those metacognitive beliefs specifically related to anger rumination, further extending the findings of previous studies (Love et al. 2022). Although several metacognitive beliefs were significantly correlated with aggressive anger expression, their direct effects were not significant. This finding expands the theoretical understanding by showing that the impacts of metacognitive beliefs about worry and control on aggressive behaviors were primarily through cognitive‐affective processes, such as anger rumination, rather than exerting a direct impact.

As a result, aggressive expression of anger was found to be a significant predictor of crash risk, supporting the notion that behavioral tendencies are closely related to driving outcomes (Demir et al. 2016; Stephens et al. 2025). Examining this relationship further demonstrates the efficacy of using the short version of Chinese DAX in predicting crash incidents in China. Such findings are crucial for designing targeted interventions that aim to improve emotional regulation for drivers, thereby enhancing road safety in China.

### 4.3 Limitations and Future Work

The present study has several limitations. One limitation of the present study concerns the relatively high attrition rate (about 60%) due to participants failing attention checks or completing the survey in an unrealistically short amount of time. Although similar exclusion strategies are increasingly recommended to ensure data quality in online studies (Douglas et al. 2023), the retained participants may possess certain characteristics, such as being more attentive and compliant when completing the survey. However, whether this also translates into differences in daily driving behaviors (e.g., being more cautious drivers) remains unclear. While the final sample showed demographic diversity and was broadly representative in terms of age, gender, and geographic distribution, we acknowledge the lack of official data on other important variables (e.g., driving mileage, personality traits), restricting to comprehensively assess the representativeness of the sample. Additionally, due to the consideration of the length of the questionnaire, traffic fines, near‐miss incidents, and minor and major crashes were not asked to report, whereas these could be used to reflect crash risk more accurately, which should be considered in future studies.

Moreover, the present study employed a cross‐sectional design, which limits the ability to draw strong causal inferences regarding the relationships among variables, despite the proposed model exhibiting sufficient predictive ability. To better understand the causal pathways between metacognition, anger rumination, driving‐related anger (e.g., aggressive anger expression), future research should consider adopting longitudinal or experimental designs, such as diary studies, driving simulations, or naturalistic driving observations. These approaches could provide more dynamic views into how drivers engage in cognitive processes when encountering anger‐evoking scenarios.

Finally, although the model shows considerable variance in predicting targeted variables, its explanatory ability to understand drivers' anger expression could be enhanced further. For example, only three dimensions were retained in the MCQ‐30 when understanding driving anger and anger rumination, and the MCQ‐30 mainly focused on the beliefs about worry and control. Thus, the newly developed measurements of metacognitive beliefs about anger processing (Moeller 2016), could be used in future work, expecting to provide more relevant information.

### 4.4 Practical Applications

The findings of this study have potential implications for road safety interventions and policy development. The validated Chinese MAD provides a reliable and valid tool for assessing trait driving anger in the contemporary Chinese driving context, enabling researchers and practitioners a practical instrument for identifying high‐anger drivers. This could be particularly useful for integrating psychological assessments into driver education and traffic management systems.

Also, the current findings provide a preliminary theoretical basis for designing targeted psychological interventions in the domain of driving anger. Specifically, some associations were observed among variables, consistent with the theoretically proposed causal framework, and it may inform the development of intervention strategies aimed at reducing crash risk and regulating aggressive anger expression in driving contexts. For instance, Cognitive‐Behavioral Interventions (CBIs), e.g., Haustein et al. (2021), have the potential to reshape drivers' maladaptive metacognitive thoughts and anger rumination, thereby helping drivers develop more adaptive coping strategies when encountering anger stimuli. Importantly, more research using either a cross‐sectional or longitudinal design is still needed to comprehensively evaluate the causal relationships between cognitive factors and driving‐related anger. This could lay a robust theoretical foundation for designing target intervention strategies.

## 5 Conclusions

This study investigated the psychometric properties of the MAD within a Chinese driving sample, also exploring its utility in linking metacognition, anger rumination and driving anger. The adapted Chinese version of the MAD comprising 23 items across three factors, demonstrated good reliability and validity. Furthermore, the MAD showed a moderate and direct relationship with anger rumination. Additionally, trait driving anger influenced anger rumination through metacognitive beliefs. Specifically, high anger drivers tended to retain more maladaptive metacognitive beliefs, such as negative thoughts about uncontrollability and danger, a heightened sense of control, and poor confidence about their memory, which is strongly associated with anger rumination. As a result, the extent of trait driving anger and anger rumination jointly influenced the aggressive forms of anger expression which further moderately predicted the crash risk, highlighting the importance of regulating the way of anger expression to prevent road trauma in China.

## Author Contributions

**Chenzhao Zhai:** conceptualization, methodology, investigation, data analysis and curation, visualization, writing – original draft, writing – review and editing, funding acquisition. **İbrahim Öztürk:** conceptualization, methodology, writing – review and editing, supervision.

## Ethics Statement

The present study was approved by the ethical committee of the University of Leeds (Ref No. BESS + FREC 2023‐0692‐869).

## Consent

Voluntary participation with written consent.

## Conflicts of Interest

The authors declare no conflicts of interest.

## Data Availability

Data will be made available on reasonable request from the corresponding author.

## Endnotes

1 The number of registered vehicles and people acquired automobile driving license respectively increased 34.2% and 25.8% between 2018 and 2022 (National Bureau of Statistics of China 2022).
